# KEAP1 Is Required for Artesunate Anticancer Activity in Non-Small-Cell Lung Cancer

**DOI:** 10.3390/cancers13081885

**Published:** 2021-04-14

**Authors:** Kristen S. Hill, Anthony McDowell, J. Robert McCorkle, Erin Schuler, Sally R. Ellingson, Rina Plattner, Jill M. Kolesar

**Affiliations:** 1 Markey Cancer Center, University of Kentucky, Lexington, KY 40536, USA; Kristen.Hill@uky.edu (K.S.H.); Rob.McCorkle@uky.edu (J.R.M.); 2 Division of Gynecologic Oncology, Department of Obstetrics and Gynecology, College of Medicine, University of Kentucky, Lexington, KY 40536, USA; amcdowell@uky.edu; 3 Department of Pathology, College of Medicine, University of Kentucky, Lexington, KY 40508, USA; Erin.E.Schuler@uky.edu; 4 Division of Biomedical Informatics, College of Medicine, University of Kentucky, Lexington, KY 40506, USA; sel228@uky.edu; 5 Department of Pharmaceutical Sciences, College of Pharmacy, University of Kentucky, Lexington, KY 40508, USA; Rina.Plattner@uky.edu; 6 Department of Pharmacy Practice and Research, College of Pharmacy, University of Kentucky, Lexington, KY 40508, USA

**Keywords:** KEAP1, NRF2, artesunate, NSCLC

## Abstract

**Simple Summary:**

The antimalarial drug artesunate also has anticancer activity. Based on what is known about how artesunate works in malaria, we hypothesized that the kelch-like ECH-associated protein 1 (KEAP1)/nuclear factor erythroid 2-related factor 2 (NRF2) pathway, which is often mutated in non-small-cell lung cancer (NSCLC), would play an important part in determining the sensitivity of NSCLC cell lines to artesunate. We treated cells with increasing doses of artesunate and showed that a cell line with an inactivating mutation in KEAP1 was less responsive to artesunate. Additionally, by modulating the KEAP1/NRF2 pathway we were able to alter the sensitivity of lung cancer cells to artesunate. Taken together, these findings demonstrate that KEAP1 is required for the anticancer activity of artesunate and support the further development of the combination of artesunate and NRF2 inhibitors to treat NSCLC, especially when patients have a mutation in the KEAP1/NRF2 pathway.

**Abstract:**

Artesunate is the most common treatment for malaria throughout the world. Artesunate has anticancer activity likely through the induction of reactive oxygen species, the same mechanism of action utilized in *Plasmodium falciparum* infections. Components of the kelch-like ECH-associated protein 1 (KEAP1)/nuclear factor erythroid 2-related factor 2 (NRF2) pathway, which regulates cellular response to oxidative stress, are mutated in approximately 30% of non-small-cell lung cancers (NSCLC); therefore, we tested the hypothesis that KEAP1 is required for artesunate sensitivity in NSCLC. Dose response assays identified A549 cells, which have a G333C-inactivating mutation in KEAP1, as resistant to artesunate, with an IC50 of 23.6 µM, while H1299 and H1563 cells were sensitive to artesunate, with a 10-fold lower IC50. Knockdown of *KEAP1* through siRNA caused increased resistance to artesunate in H1299 cells. Alternatively, the pharmacological inhibition of NRF2, which is activated downstream of KEAP1 loss, by ML385 partially restored sensitivity of A549 cells to artesunate, and the combination of artesunate and ML385 was synergistic in both A549 and H1299 cells. These findings demonstrate that KEAP1 is required for the anticancer activity of artesunate and support the further development of NRF2 inhibitors to target patients with mutations in the KEAP1/NRF2 pathway.

## 1. Introduction

Artesunate, first discovered in 1972 by the Chinese scientist Tu Youyou, is a water-soluble semi-synthetic derivative of artemisinin [1]. This Nobel Prize-winning medication has become the WHO-recommended first-line treatment for severe malaria [2]. Artemisinin and its derivative, artesunate, are obtained as extracts from *Artemisia annua*, or the sweet wormwood plant [3].

Artesunate has a sesquiterpene lactone with a peroxide bridge that is responsible for its biological activity. In addition to antimalarial activity, artesunate has potential anticancer effects and proposed mechanisms include induction of apoptosis, inhibition of angiogenesis, inhibition of hypoxia-inducible factor-1α (HIF-1α) activation, and direct DNA injury. Generation of reactive oxygen species (ROS) is common among these mechanisms, specifically by generating hydroxyl radicals [4]. Artesunate has been clinically investigated in combination with cisplatin and vinorelbine in patients with advanced non-small-cell lung cancer (NSCLC). In a small study, both the disease control rate and the time to tumor progression were significantly improved for the artesunate and chemotherapy group when compared to the chemotherapy alone group with no significant differences in toxicity [5].

The use of artesunate in the treatment of malaria is limited by the development of resistance mediated by kelch13 mutations in the causative organism, *Plasmodium falciparum* [6,7,8]. The three most common mutations associated with resistance are C580Y, R539T and Y493H and are found in the propeller region of the gene and mutations are thought to destabilize and inactivate the protein. Kelch-like ECH-associated protein 1, (KEAP1) is the human ortholog of kelch13.

KEAP1 binds to nuclear factor erythroid 2-related factor 2 (NRF2), a key regulator of cellular response to oxidative stress, in the cytoplasm. Under normal physiological conditions, KEAP1 forms a complex with NRF2 and CUL3 (Cullin-3), which facilitates the ubiquitination and subsequent proteasomal degradation of NRF2 [9,10,11]. In response to oxidative stress, KEAP1 cysteine residues are oxidized, leading to conformational changes that cause release of NRF2, allowing for NRF2 to translocate to the nucleus, and regulate the transcriptional activation of antioxidant response genes, including heme oxygenase-1 (*HO-1*) and NAD(P)H quinone dehydrogenase 1 (*NQO-1*) [10]. Mutations in this pathway occur in approximately 30% of NSCLC and are of considerable clinical interest. In NSCLC cell lines, overexpression of wild-type, but not inactivating mutant, *KEAP1* results in reduced colony formation in soft agar, decreased cell migration, and reduced growth of tumors in subcutaneous xenografts [9]. Additionally, overexpressing wild-type *KEAP1* reduces the expression of NRF2 protein and the expression of transcriptional targets of NRF2 including *HO-1* and *NQO-1* [9]. Genetically engineered mouse models have also demonstrated that NRF2 activation or deletion of *Keap1* accelerates *Kras^G12D^* driven lung tumorigenesis [12,13,14]. When compared to individuals without mutations in this pathway, patients with mutations in the KEAP1/NRF2 pathway have significantly shorter progression-free survival and overall survival [15], less benefit from epidermal growth factor receptor (EGFR) inhibitors [16], insensitivity to chemotherapy [17], and increased metastasis [18]. These studies show that inactivating *KEAP1* mutations promote tumor growth and migration through activating NRF2 mediated transcription of antioxidant response genes like *NQO1* and mutations in *KEAP1/NRF2* are potential drivers of clinical treatment resistance to a variety of agents.

Given the frequency and clinical significance of mutations in this pathway, effective treatment strategies for *KEAP1/NRF2*-mutated cancer are needed. The purpose of this research is to understand the role of KEAP1 in artesunate sensitivity and develop potentially effective combination strategies.

## 2. Results

### 2.1. Artesunate Sensitivity Varies Across a Small Panel of NSCLC Cell Lines

**Figure 1:**
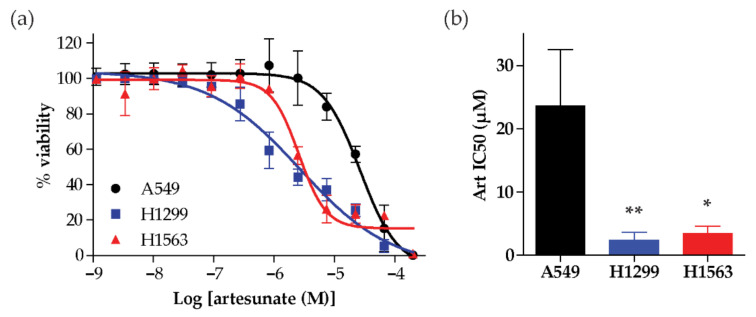
Artesunate sensitivity across a panel of non-small-cell lung cancer (NSCLC) cell lines. (**a**) Cells were treated with a serially diluted concentrations of artesunate for 96hr. Each cell lines was normalized to cells treated with 0.1% DMSO (dimethyl sulfoxide) as a control and is graphed as the mean ± SD, *n* = 6. (**b**) The mean IC50 of artesunate (Art) in each cell line is graphed ± SD. *p*-values were calculated using a two-tailed *t*-test (* *p* = 0.0290 or ** *p* = 0.0007 compared to A549).

We first investigated the effect of artesunate on three NSCLC cell lines (A549, H1299, and H1563) by assessing cell viability, using a CellTiter-Glo 2.0 assay, after treatment with increasing concentrations of artesunate for 96 h. The cell lines separated as either sensitive or resistant with a 10-fold difference in IC50 values for artesunate, (Figure 1a). A549 cells were resistant to artesunate, with a mean IC50 of 23.63 µM ± 8.886 µM from three independent experiments, while H1299 and H1563 cells were sensitive to artesunate, with mean IC50s of 2.36 µM ± 1.275 µM and 3.43 µM ± 1.190 µM, respectively, (Figure 1b).

### 2.2. Generation of Reactive Oxygen Species

**Figure 2:**
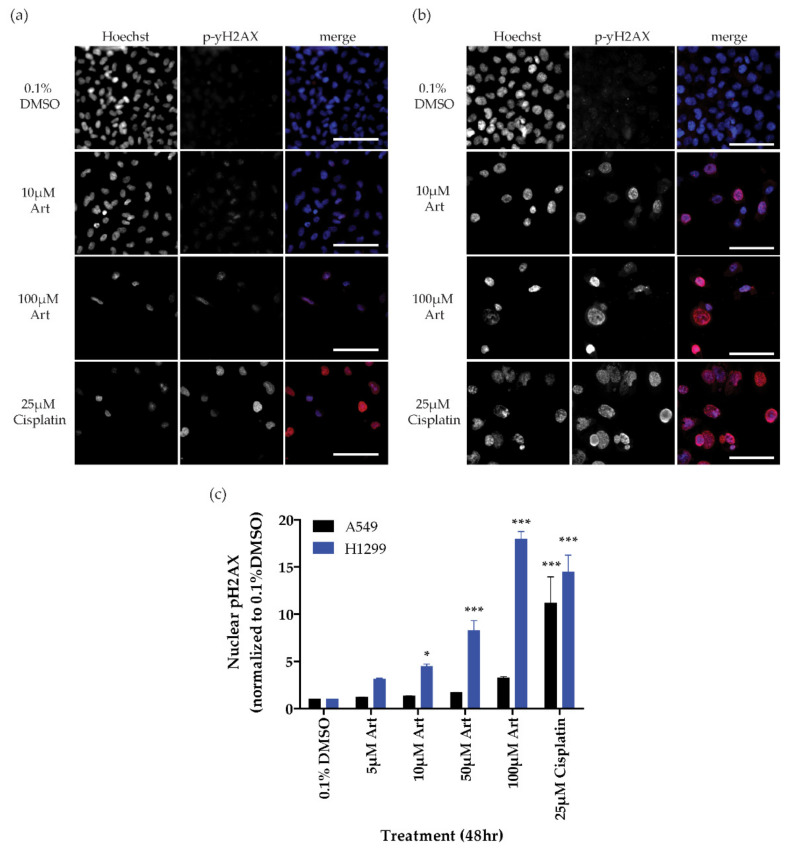
Dose-dependent induction of DNA damage by artesunate in NSCLC cell lines. Representative images for A549 cells (**a**) and H1299 cells (**b**) treated for 48 h with the indicated drug concentration (bar = 50 µm). (**c**) Quantification of nuclear pH2AX staining. Graphed as mean fold change in nuclear fluorescent intensity signal normalized to 0.1% DMSO per cell ± SD. *p*-values calculate by one-way ANOVA for each cell line with Dunnett’s multiple comparison test comparing to matched 0.1% DMSO control (* *p* < 0.05, *** *p* < 0.001).

Artesunate’s primary mechanism of action against malaria is to generate reactive oxygen species (ROS) through a reaction between the endoperoxide bridge within artesunate and heme iron in the malaria plasmodium [4,8]. Within cancer cells, the generation of ROS can cause DNA damage through double-strand breaks which can be assessed by immunofluorescent staining for phosphorylated histone H2AX (pH2AX) [19,20]; therefore, we assessed the ability of artesunate to induce DNA damage in A549 (resistant) and H1299 (sensitive) NSCLC cell lines in a dose-dependent manor. Treatment with 25 µM cisplatin was used as a positive control for DNA damage while 0.1% DMSO was used as a negative/vehicle control. The mean nuclear pH2AX signal intensity for each treatment was normalized to the matched 0.1% DMSO control and plotted as the ratio of treatment/0.1% DMSO (dimethyl sulfoxide) ± SD, (Figure 2). In both A549 and H1299 cells, cisplatin induced significantly more (*p* < 0.001) pH2AX staining than what was observed in matched 0.1% DMSO-treated control cells as determined by a one-way ANOVA with Dunnett’s multiple comparison test for each cell line). Specifically, 25 µM cisplatin resulted in a 11.17 ± 2.79-fold increase in pH2AX staining in A549 cells and a 14.45 ± 1.82-fold increase in H1299 cells. In contrast, in A549 cells, increasing concentrations of artesuante, when compared to 0.1% DMSO-treated controls, did not cause significantly increased DNA damage (5 µM: 1.19 ± 0.05, 10 µM: 1.35 ± 0.01, 50 µM: 1.71 ± 0.02, 100 µM: 3.27 ± 0.13), even at the highest concentration of artesunate. Alternatively, in the artesunate sensitive H1299 cells, significant DNA damage (*p* < 0.05) was observed following treatment with as little as 10 µM artesunate. Specifically, 5 µM artesunate resulted in a 3.14 ± 0.09-fold increase in pH2AX staining, 10 µM artesunate produced an increase of 4.47 ± 0.26 fold, 50 µM produced an increase of 8.25 ± 1.08, and 100 µM artesunate increased pH2AX staining by 17.97 ± 0.79-fold compared to 0.1% DMSO control-treated cells.

### 2.3. Artesunate Sensitivity Is Dependent on KEAP1 in NSCLC

**Figure 3:**
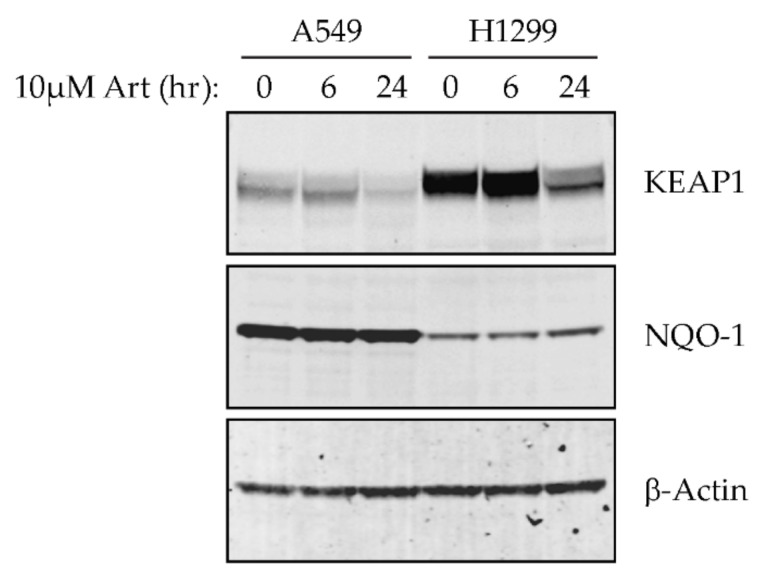
Artesunate treatment induces changes in kelch-like ECH-associated protein 1 (KEAP1)/nuclear factor erythroid 2-related factor 2 (NRF2) pathway protein expression in a time-dependent manor in A549 and H1299 NSCLC cell lines. Cells were treated with 10 µM artesunate for 0, 6, or 24 h and levels of KEAP1 and NQO-1 (NAD(P)H quinone dehydrogenase 1) proteins were assessed by Western blot. In both cell lines, KEAP1 protein levels are reduced following treatment with artesunate. The original blots can be found at Figure S5.

A549 cells harbor a G333C mutation in KEAP1 [10], while both H1299 and H1563 cells are wild type for KEAP1 and NFE2L2 (the gene encoding NRF2), which could account for the innate resistance of A549 cells to artesunate. Because artesunate is known to increase cellular ROS and the KEAP1/NRF2 pathway is a master regulator of cellular response to ROS, we first assessed whether treatment of A549 (resistant) or H1299 (sensitive) cells with artesuante altered the protein expression of either KEAP1 or NQO-1. NQO-1 is a well established transcriptional target of NRF2 as is used as a proxy to assess NRF2 transcriptional activity by Western blot [9,10,11,21,22]. Cells were treated with 10 µm artesuante for 0, 6, or 24 h prior to lysate collection and a Western blot for KEAP1, NQO-1, and B-ACTIN was performed. The KEAP1 antibody used for Western blotting was generated using a peptide present in both the wild-type and G333C mutant KEAP1 and therefore is able to detect both the wild-type and mutant forms from the cell lines tested. As seen in Figure 3, A549 cells had reduced basal expression of KEAP1 and increased basal expression of NQO-1 compared to H1299 cells, which is as expected since A549 cells harbor an inactivation mutation in KEAP1. Treatment of both cell lines with artesunate for 24 h resulted in decreased expression of KEAP1. Additionally, in H1299 cells, a slight but detectable increase in NQO-1 protein is observed after 24 h treatment with 10 µM artesunate.

**Figure 4:**
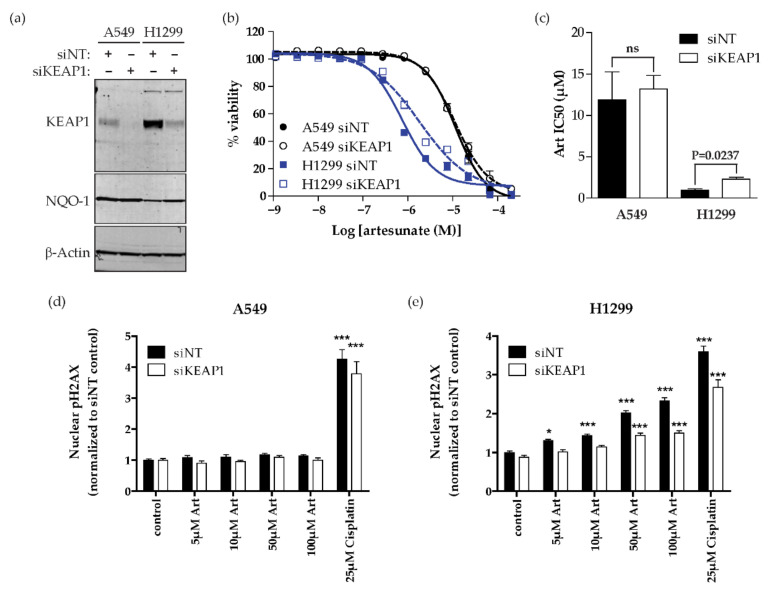
Knocking down *KEAP1* increases resistance to artesunate in H1299 cells (**a**) Western blot analysis for KEAP1, NQO-1 and ß-Actin shows reduced KEAP1 protein in siKEAP1-treated cells 48 h after transfection. (**b**) Artesunate dose–response curves after transfection with non-targeting (siNT—solid) or KEAP1 (siKEAP1—dashed) siRNA. Data was normalized to 0.1% DMSO and is plotted as mean ± SD, *n* = 4. (**c**) The mean IC50 of artesunate in each cell line transfected with non-targeting (siNT) siRNA (solid) or siKEAP1 (open) is graphed ± SD. *p*-values were calculated using a two-tailed *t*-test. (**d**,**e**) Quantification of nuclear pH2AX staining in A549 (**d**) and H1299 (**e**) following transfection with siNT or siKEAP1. DNA damage was assessed by nuclear pH2AX staining after 24 h treatment with 0.1% DMSO (control), the indicated concentration of artesunate, or 25 µM cisplatin. Nuclear pH2AX staining is was normalized to cells transfected with siNT and treated with DMSO control and is plotted as mean fold change ± SD. *p*-values calculate using a two-way ANOVA comparing each artesunate concertation to the matched control after normalization (* *p* < 0.05; *** *p* < 0.001).

Since KEAP1 expression is reduced by artesunate in both cell lines, to determine whether KEAP1 expression regulates sensitivity to artesunate, we used siRNA to knockdown KEAP1 in both H1299 (sensitive) and A549 (resistant) cells. The siRNA used for this experiment was a pool of four sequences that did not overlap with the mutation site found in A549 cells; therefore, they were predicted to knockdown both wild-type and mutant KEAP1 mRNA and thus reduce KEAP1 protein levels. Knocking down mutant KEAP1 in A549 cells did not affect NQO-1 expression or response to artesunate (Figure 4a). However, knocking down KEAP1 in H1299 cells resulted in increased NQO-1 expression, indicating activation of the NRF2 antioxidant transcriptional pathway. Additionally, knocking down KEAP1 in H1299 cells increased the mean IC50 of artesunate from 0.98 µM in siNT-transfected cells to 2.30 µM in siKEAP1-transfected cells, (Figure 4b,c). Next we assessed the effect of *knocking down KEAP1* on artesuante induced DNA damage. In cells with an inactivating KEAP1 mutation, A549, knocking down KEAP1 had no effect on artesuante induced DNA damage. In these cells, only treatment with 25 µM cisplatin resulted in a significant increase in DNA damage compared to matched control cells with a fold change in pH2AX staining of 4.255 ± 0.759 in siNT-transfected cells and 3.791 ± 0.957 in siKEAP1-transfected cells (Figure 4d and Figure S1). In H1299 cells, knocking down KEAP1 resulted in a significant decrease in artesunate induced DNA damage compared to cells treated with non-targeting control siRNA (siNT) when cells are treated with 50 µM and 100 µM artesunate or with 25 µM cisplatin (Figure 4e). Specifically, 50 µM artesunate resulted in a 2.203 ± 0.170-fold increase in DNA damage in siNT-transfected cells while only a 1.440 ± 0.186-fold increase in DNA damage was observed in siKEAP1-transfected cells when compared to matched control-treated cells. Treatment with 100 µM artesunate and 25 µM cisplatin also resulted in significantly less DNA damage in KEAP1 knockdown cells with 1.502 ± 0.168- and 2.678 ± 0.580-fold nuclear pH2AX staining, respectively compared to 2.238 ± 0.251 and 3.599 ± 0.414 for siNT-transfected cells. Therefore, dysregulation of the NRF2 pathway through decreased expression of KEAP1 or loss of function KEAP1 mutations causes increased resistance to artesunate in NSCLC cell lines.

### 2.4. NRF2 Inhibition Sensitizes Resistant NSCLC Cells (KEAP1 Mutant) to Artesunate

**Figure 5:**
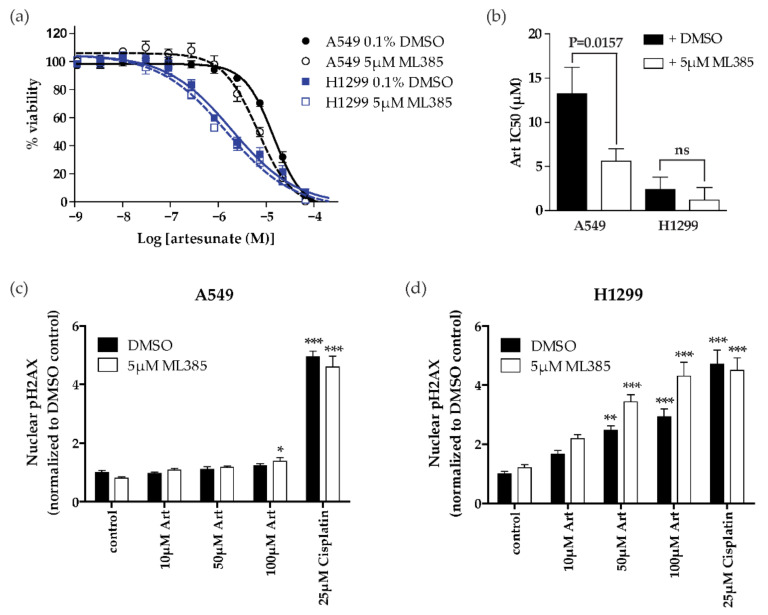
Pharmacological inhibition of NRF2 sensitizes A549 cells to artesunate (**a**) Artesunate was added to media with 0.1% DMSO (solid) or 5 µM ML385 (dashed) and artesunate dose response data was normalized to DMSO or 5 µM ML385 alone and is graphed as mean ± SD, *n* = 6. (**b**) The mean IC50 of artesunate in each cell line with 0.1% DMSO (solid) or 5 µM ML385 (open) is graphed ± SD. *p*-values were calculated using a two-tailed *t*-test. (**c**,**d**) Quantification of nuclear pH2AX staining in A549 (**c**) and H1299 (**d**) cells that were pretreated for 24 h with 0.03% DMSO or 5 µM ML385 followed by the addition of the indicated concentration of artesunate for an additional 24 h. Graphed as mean fold change ± SD in nuclear fluorescent intensity signal normalized to vehicle control cells pretreated with 0.03%. P-values calculate using a one-way ANOVA with Dunnett’s multiple comparison test comparing each artesunate concertation to the matched control after normalization (** *p* < 0.01; *** *p* < 0.001).

Since activation of the NRF2 pathway occurs downstream of KEAP1 loss, we next investigated whether the pharmacological inhibition of NRF2 could sensitize resistant A549 cells to artesunate. We used ML385, a small molecule shown to bind NRF2 and inhibit its function as a transcription factor by preventing DNA binding [21], to test the effect of NRF2 inhibition on artesunate sensitivity in both A549 (resistant) and H1299 (sensitive) cells. Prior to treating cells with a combination of artesuante and ML385 we first assessed the effect of ML385 alone on A549 and H1299 cells. ML385 had minimal effect on cell viability as a single agent in either cell line and there was no significant difference in IC50 observed between the two cell lines tested, (Figure S2). Next A549 and H1299 cells were treated with increasing concentrations of artesunate alone or in combination with 5 µM ML385. In order to control for any effect of the addition of 5 µM ML385 had on the cells directly the percent viability of cells treated with artesunate alone was normalized to a DMSO (vehicle) control while cells treated with artesunate + 5 µM ML385 were normalized to cells treated with 5 µM ML385. The addition of 5 µM ML385 caused the IC50 to shift from 13.3 µM in A549 cells treated with artesunate plus 0.1%DMSO to 5.60 µM in cells treated with artesunate plus 5 µM ML385 indicating that NRF2 inhibition sensitized these cells to artesunate, (Figure 5a,b). In the H1299, artesunate sensitive, cell line there was also a slight leftward shift in IC50 (2.35 µM to 1.19 µM in combination with ML385); however, this change was not statistically significant, (Figure 5b). Next we assessed the ability of 5 µM ML385 to enhance artesunate induced DNA damage in A549 and H1299 cells. For this assay, cells were pretreated for 24 h with 5 µM ML385 or 0.03% DMSO. The next day, fresh media was prepared with 0.03% DMSO or 5 µM ML385 with alone or with 10 µM, 50 µM, or 100 µM artesunate or 25 µM cisplatin and cells were incubated for an additional 24 h. Nuclear pH2AX staining was quantified from three independent experiments and was normalized to control cells which were pretreated with 0.03% DMSO (Figure 5c,d and Figure S3). ML385 alone did not increase DNA damage in A549 (resistant) or H1299 (sensitive) cells. Cotreatment with ML385 and artesunate resulted in statistically significant increase in DNA damage in A549 (resistant) cells treated only at the highest dose of100 µM artesunate when compared to cells treated with 5 µM ML385 alone. Specifically 100 µM artesunate plus 5 µM ML385 resulted in 1.390 ± 0.327-fold DNA damage compared to 0.806 ± 0.142 in cells treated with 5 µM ML385 alone. In H1299 cells, cotreatment with artesunate and ML385 resulted in significant increases in DNA damage with both 50 µM and 100 µM artesunate (3.431 ± 0.685 and 4.309 ± 1.309, respectively), but not in cells treated with 10 µM artesunate (2.187 ± 0.401).

### 2.5. Artesunate and NRF2 Inhibition Are Synergistic

**Figure 6:**
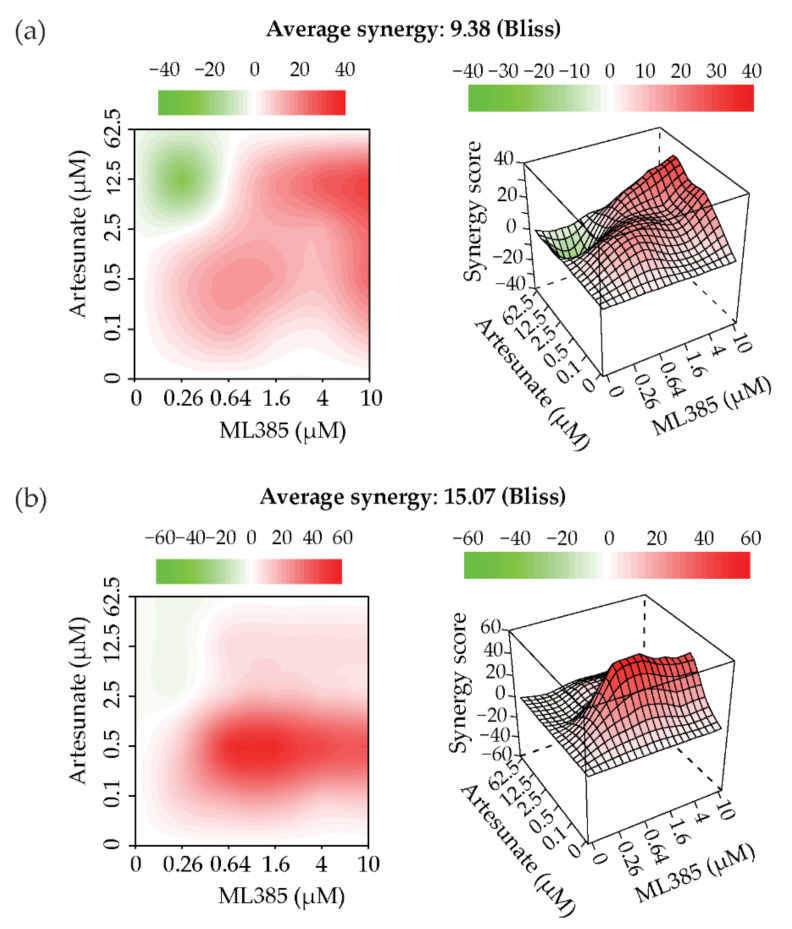
Artesunate is synergistic with ML385 (Nrf2 inhibitor). (**a**) Graphic representation of the Bliss independence model of synergy scoring as calculated by using a 6 × 6 dose–response matrix in A549 (**a**) and H1299 (**b**) cells. Red color indicates synergy, while green indicates antagonism between the drug combinations tested.

To better understand the effectiveness of combining artesunate with the NRF2 inhibitor, ML385, we next tested whether this drug combination was synergistic. A synergistic drug interaction is when one or both of the drugs used enhances the effectiveness of the partner drug; therefore, allowing lower concentrations of each drug to be used to achieve a clinical benefit in patients. To assess synergy, we utilized a 6 × 6 checkerboard method coupled with both Bliss and ZIP models to assess drug combinations. The Bliss independence model has been one of the standards for assessing drug combinations since it was introduced by in 1939 [23], while the ZIP model was developed in 2015 to address some of the limitations of the Loewe and Bliss models [24]. The output of both of these models is a synergy score in which a negative score indicates antagonism while a positive score indicates synergy. When artesunate and ML385 were combined in A549 (artesunate resistant) cells, the Bliss score was 9.38 (Figure 6a), while the ZIP score was 8.744 (Figure S4a). In the H1299 (artesunate sensitive), the Bliss score was 15.07 (Figure 6b), and the ZIP score was 16.593 (Figure S4b). This shows that artesunate and ML385 are synergistic in NSCLC cell lines independent of KEAP1 mutational status. In addition, synergy is observed at clinically achievable plasma concentrations (0.1–0.5 µM) concentrations of artesunate.

## 3. Discussion

The prevalence of mutations in either *KEAP1* or *NFE2L2* (the gene encoding NRF2) is approximately 30% overall in NSCLC, with *KEAP1* mutations occurring primarily in adenocarcinoma and *NFE2L2* in squamous cell carcinoma [25,26]. Mutations are typically gain of function in NRF2, loss of function in KEAP1, and are mutually exclusive [22]. NRF2 mutations may enable the transcription factor to evade KEAP1-mediated repression and KEAP1 loss-of-function mutations likely make KEAP1 unable to bind to and regulate NRF2. KEAP1/NRF2 mutational status is associated with poor prognosis and chemotherapeutic resistance in NSCLC [11]. The prevalence of mutations in the KEAP1/NRF2 pathway in NSCLC and their role in tumorigenesis and progression underscore the need to investigate potential therapies in the context of KEAP1/NRF2 mutations. This is even more important when the purposed mechanism of action for the drug of interests involves ROS generation, like artesunate.

In *Plasmodium falciparum,* mutations in the propeller regions of kelch-13, which is homologous to the region of human KEAP1 that is responsible for the association with NRF2, are implicated in artesunate resistance [6,8]. This is similar to the observations in this study showing that KEAP1 mutational status plays a role in determining the sensitivity of NSCLC cell lines to artesunate. Specifically, we show that the A549 cell line, which has an inactivating mutation in KEAP1 [10], is inherently resistant to artesunate. In contrast, both H1299 and H1563 are sensitive to artesunate and have wild-type KEAP1 (Figure 1). The importance of KEAP1 to artesunate sensitivity was further demonstrated by the inability of artesunate to induce significant DNA damage in A549 cells while low micromolar concentrations of artesunate resulted in significant DNA damage in H1299 cells (Figure 2). Using siRNA, we demonstrated that we are able to knockdown both the mutant and wild-type forms of KEAP1 in A549 and H1299 cells, respectively. *KEAP1* knockdown in the A549 cell line, which has an inactivating KEAP1 mutation, has little effect on sensitivity to artesunate and did not impact artesunate induced DNA damage. Alternatively, in the KEAP1 wild-type cell line, H1299, knocking down KEAP1 makes the cell line significantly more resistant to artesunate and inhibited artesunate induced DNA damage when compared to cells transfected with a non-targeting control siRNA sequence, suggesting that KEAP1 is critical to artesunate sensitivity. It is not unexpected that knocking down mutant KEAP1 protein in A549 cells has little to no effect on their sensitivity to artesunate because the protein being lost is already inactivated by the mutation; therefore, reducing the expression of mutant KEAP1 protein would not alter the stability or transcriptional activity of NRF2 which is responsible for the a high basal antioxidant response in A549 cells.

Having identified KEAP1 as an important regulator of artesunate resistance in NSCLC cell lines the next step was to determine whether there was a druggable target downstream of KEAP1 that could be targeted in combination with artesunate to prevent or overcome resistance to artesunate. The most logical target to investigate is NRF2 because it is a direct binding partner of KEAP1 and is currently being investigated as a therapeutic target in a number of disease states. In the United States, there is one marketed NRF2 activator, dimethyl fumarate (DMF) which is FDA approved for the treatment of multiple sclerosis [27,28] and sulforaphane is currently in phase 2 development for the treatment of breast cancer [29]. Another approach in preclinical development is the targeting of the interaction between NRF2 and small musculoaponeurotic fibrosarcoma oncogene homologue (smAF) proteins, which is required for the transcriptional activity of NRF2. ML385 is a thiazole–indoline which binds to the carboxy terminal domain of NRF2 and interferes with the formation of the NRF2–smAF protein heterodimer that is required for antioxidant response element (ARE) gene expression [21]. A number of NRF2–KEAP1 protein–protein interaction inhibitors are also in preclinical development. Currently, NRF2 inhibitors are being developed as single agents, a strategy that is likely to be ineffective in the many patients with mutations in KEAP1 or NRF2. Since dual inhibition of proteins in a signaling pathway with a mutually exclusive mutation pattern can result in synthetic lethality, we next used 5 µM ML385 to inhibit NRF2 in both KEAP1 wild-type and mutant cell lines. While NRF2 inhibition had less effect in the already sensitive KEAP1 wild-type cell line, in the KEAP1 mutant cell line, inhibition of NRF2 sensitized the cell line to artesunate (Figure 5). This is the predicted result based on the hypothesis that KEAP1 mutant cell lines, like A549 cells, have increased stability and thus transcriptional activity of NRF2. Increased basal NRF2 activity has the potential to make these cells lines more responsive to NRF2 inhibition. In our studies, the difference in sensitivity of A549 and H1299 cells to ML385 (NRF2 inhibition) was only significant when cells were also treated with artesunate, Figure 5 and Supplemental Figure S2, which could indicate cooperation between artesunate and ML385 in KEAP1 mutant lung cancer cells. However, artesunate was able to induce significant DNA damage at lower concentrations when cells were also treated with ML385 compared to cells that had been treated with a vehicle control in both A549 and H1299 cells. Therefore, we next evaluated the optimal synergistic combinations of ML385 and artesunate in both A549 and H1299 cells. We utilized two models to assess the combination of artesunate with ML385; specifically, the Bliss independence model [23] and the zero interaction potency (ZIP) model [24] which was developed to overcome limitations of the Loewe and Bliss models. The output from both models is a synergy score, which is centered at zero; therefore, the more positive the score, the more synergistic the combination, while the more negative the score, the more antagonistic the combination. As shown in Figure 6, we demonstrate artesunate and ML385 as a synergistic combination, regardless of *KEAP1* mutation status which is consistent with our data that treatment with the combination of artesunate and ML385 induced significantly more DNA damage, in both cell lines tested, when compared to treatment with artesunate alone. It is possible the observed synergy in KEAP1 wild-type cells was due to the drugs being used at lower concentrations in the synergy experiment than what was used to determine the IC50. In both the A549 and H1299 cell lines, synergy between artesunate and ML385 was observed at drug concentrations in the submicromolar range which are likely clinically achievable [30,31] and thus have the potential to be observed in patients.

To our knowledge, this is the first study to show that treatment with artesunate results in reduced levels of KEAP1 protein and that *KEAP1* mutational status is a key factor in cellular response to artesunate. Future studies will be needed to determine the mechanism of how artesunate alters KEAP1 protein expression. One limitation of the current study is that we only measured cell viability and DNA damage to determine whether artesunate had anticancer activity in the cell lines tested and did not directly assess ROS production or apoptosis. One reason for this is that it has been well established in the literature that the primary mechanism of action for artesunate is the induction of ROS [4,8,32,33]. Additionally, the focus of this study was assessing whether *KEAP1* mutation was associated with resistance to artesunate, similar to what has been observed with mutations in kelch-13 in malaria, and whether we could identify a drug that could be used in combination with artesunate to overcome this resistance. Ultimately, this study identified that the combination of artesunate with the NRF2 inhibitor, ML385, was able to overcome cellular resistance to artesunate mediated by *KEAP1* mutation. Further studies, including in vivo assessment of both efficacy and safety, are needed before this drug combination can be moved into clinical trial, but the initial preclinical studies shown in this study are promising.

In summary, like in malaria, where kelch13 mutations cause resistance to artesunate, a NSCLC cell line with a *KEAP1* inactivating mutation is resistant to artesunate. This resistance to artesunate can be overcome with the addition of a synergistic small-molecule NRF2 inhibitor. In addition, given that KEAP1 mutations are common in NSCLC and are associated with poor prognosis and chemoresistance, the combination of artesunate and an NRF2 inhibitor may be a rational and effective combination therapy for future study.

## 4. Materials and Methods

### 4.1. Cell Lines and Reagents

A549 (ATCC: CCL-185), H1299 (ATCC: CRL-5803), and H1563 (ATCC: CRL-5875) NSCLC cell lines were purchased directly from ATCC. All cell lines were initially expanded and low passage numbers aliquots were frozen back to ensure experiments were conducted in cell lines with similar passage numbers. Cell lines were screened for mycoplasma at regular intervals, including when cell lines were frozen back. All cells were grown in RPMI 1640 (Lonza, Basel, Switzerland: 12-167F) with 10% Fetal Bovine Serum (Sigma-Aldrich, St. Louis MO, USA: F0926), Penicillin/Streptomycin (Gibco, Waltham, MA, USA: 15140-122), and 2 mM Glutamax (Gibco: 35050-061) and were maintained in a 37 °C humidified incubator with 5% CO_2_. Purified artesunate (HY-N0193) was purchased from MedChem Express (Monmouth Junction, NJ, USA) and ML385 (SML1833) was purchased from Sigma-Aldrich.

### 4.2. Drug Response Assays

Cells are seeded into white-walled 96-well plates at 2500 cells (A549 and H1299) or 4000 cells (H1563) per well in 100 µL of complete growth media and allowed to adhere for 24 h at 37 °C with 5% CO_2_. After 24 h, artesunate is serially diluted 1:3 in DMSO to obtain 12 drug stocks in 100% DMSO; subsequently, each stock is diluted 1:1000 in complete media so the final concentration of DMSO is 0.1%. Growth media is aspirated off the cells and replaced with media containing the diluted artesunate, with each drug concentration being tested on duplicate wells; additionally, triplicate wells receive media with only 0.1% DMSO and serve as untreated controls. Cells are incubated with drugs for 96 h prior to using CellTiter-Glo 2.0 (Promega, Madison, WI, USA: G9243) to assess cell viability. Data is presented as the percent viability of treated cells normalized to 0.1% DMSO-treated control cells. GraphPad Prism (version 5.01) was used to fit a dose response curve (four parameter log-logistic model) to the data and to calculate IC50 values. For studies assessing artesunate in combination with ML385, artesunate drug stocks were diluted in media containing 5 µM ML385 and the matched control wells were treated with 5 µM ML385.

### 4.3. DNA Damage Assay

A549 and H1299 cells are seeded into black-walled µClear 96-well plates (ThermoScientific, Waltham MA, USA: 165305) at a density of 3000 cells per well in 100 µL of complete growth media and allowed to adhere for 24 h at 37 °C with 5% CO2. Subsequently, media was removed and replaced with complete media containing 5 µM, 10 µM, 50 µM, 100 µM Artesunate, 0.1% DMSO as a negative control or 25 µM Cisplatin (Tocris Bioscience, Minneapolis MN, USA: 2251) as a positive control. Cells were incubated with drugs for 48 h and then fixed for 15min at RT in 4% paraformaldehyde (Alfa Aesar, Tewksbury, MA, USA: J61899). Following fixation, cells were permeabilized with 0.25% Triton X-100 (Alfa Aesar: A16046) for 15 min and blocked in 0.1% bovine serum albumin (BSA) for 1hr. DNA damage was assessed by immunofluorescence staining for phosphorylated histone H2AX (pH2AX) using the HCS DNA Damage Kit (Invitrogen, Waltham MA, USA: H10292). Cells were imaged using the CellInsight CX7 High Content Analysis Platform (ThermoScientific: CX7A1110) and quantification of nuclear pH2AX signal was performed using the HCS Studio software (ThermoScientific). Statistical analysis of pH2AX signal was performed on GraphPad Prism (version 5.01). The effect of the combination treatment of artesunate and ML385 on DNA damage was also assessed using the HCS DNA Damage Kit. The day after cells were seeded, as above, they were treated with DMSO or 5 µM ML385 for 24 h. The next day, the media was aspirated and fresh media containing 10 µM, 50 µM, 100 µM artesunate, 0.1% DMSO as a negative control or 25 µM Cisplatin with or without fresh 5 µM ML385 was added to the cells. After 24 h, the cells were fixed with 4% paraformaldehyde and DNA damage was assessed as described above.

### 4.4. Western Blotting

A549 and H1299 cells were treated with 10 µM artesunate for 0, 6, or 24 h prior to being lysed in RIPA buffer (Pierce, Waltham, MA, USA: 89900) containing Halt Protease and Phosphatase Inhibitor Cocktail (ThermoScientific: 78441) and Benzonase Nuclease (Sigma: E1014), incubated on ice for 10min, and cleared by centrifugation. Protein concentrations were determined using a BCA Protein Assay (Pierce: 23227) and 40 µg total protein was loaded onto a NuPAGE 4–12% Bis-Tris Gel (Life Technologies, Waltham MA, USA: NP0321BOX) for electrophoresis prior to transfer to a PVDF membrane (Invitrogen: LC2005) for blotting. Antibodies against KEAP1 (Cell Signaling, Danvers, MA, USA: 8047S) and NQO-1 (Cell Signaling: 3187S) were purchased from Cell Signaling, while antibodies to ß-ACTIN were purchased from R&D Systems (MAB8929). IRDye-conjugated secondary antibodies were purchased from LI-COR (925-32213 and 925-68070) and Westerns blots were imaged using a LI-COR Odyssey imaging system.

### 4.5. siRNA Knockdown

Two wells of a 6-well plate were seeded with 2 × 10^5^ A549 or H1299 cells per well and allowed to adhere overnight before transfection with 5 nM siGENOME Control pool non-targeting #2 (Dharmacon, Boulder CO, USA: D-001206-14-5) or siGENOME SMARTpool siRNA targeting human *KEAP1* (GGACAAACCGCCUUAAUUC; CAGCAGAACUGUACCUGUU; GGGCGUGGCUGUCCUCAAU; CGAAUGAUCACAGCAAUGA) purchased from Dharmacon (M-012453-00-0005). After 24 h, cells were seeded into a 96-well plate at 2500 cells per well or were seeded into 60 mm tissue culture plates to assess knockdown efficiency by Western blot. Then 48 h after initial transfections, cells seeded into 96-well plates were treated as detailed above (Drug response assays) to assess the effect of KEAP1 knockdown on drug sensitivity, while the 60 mm plates were harvested in RIPA buffer for Western blotting (detailed above in Section 4) to determine knockdown efficiency. To determine whether knocking down *KEAP1* altered artesunate’s ability to induce DNA damage in NSCLC cells, cells were transfected with 5 nM non-targeting control or siRNA targeting human *KEAP1*. The next day, 6000 cells per well were seeded in complete growth media in a black-walled clear bottom 96-well plate and a DNA damage assay was performed as described above, *4.3. DNA Damage Assay*.

### 4.6. Synergy

To assess drug interactions, drug response assays were preformed similarly to the method above; however, a 6 × 6 matrix design was used to assay pairs of drugs alone and in combination with five serially diluted concentrations of each drug. Cell viability was assessed following a 96 h treatment using CellTiter-Glo 2.0. Each well was normalized to untreated control cells which were grown in media with 0.2% DMSO and the percentage of viable cells was determined. R statistical software, specifically the snergyfinder package (version 1.10.4) [34], was used to generate a synergy score using the Bliss independence model [23] and the Zero Interaction Potency (ZIP) model [24].

### 4.7. Statistical Analysis

To assess whether the difference in artesunate IC50 values were statistically significant, the IC50 values calculated from multiple independent experiments along with the standard error for each experiment were graphed using GraphPad Prism 5. A two-tailed *t*-test was used to determine whether the mean IC50 between two different cell lines or between treated and control cells were statistically different. *p*-values are reported in the figure legends and results with a *p*-value less than 0.05 indicating a statistical significant difference. Differences in DNA damage was determined for each cell line using a one-way ANOVA followed by Dunnett’s multiple comparison test to compare each treatment to 0.1% DMSO control for cells treated with artesunate alone or in combination with 5 µM ML385. A two-way ANOVA with Bonferroni post test was utilized to assess DNA damage following 24 h artesunate treatment in cells transfected with siRNA.

## 5. Conclusions

In conclusion, siRNA and small-molecule inhibitor studies show that inactivating *KEAP1* mutations in NSCLC, like mutation in kelch13 in *Plasmodium falciparum*, result in resistance to artesunate. This mutant *KEAP1* mediated resistance to artesunate can be overcome by combining artesunate with a synergistic small-molecule NRF2 inhibitor. This observation coupled with the prevalence of KEAP1/NRF2 pathway mutations in NSCLC lends support to further investigation of artesunate and an NRF2 inhibitor as a novel drug combination for the treatment for patients with NSCLC.

## Data Availability

The data presented in this study are available in this article (and Supplementary Material).

## Supplementary Materials

The following are available online at https://www.mdpi.com/article/10.3390/cancers13081885/s1, Figure S1: Representative images of DNA damage in siRNA-transfected cells; Figure S2: ML385 effect on cell viability as a single agent; Figure S3: Representative images of DNA damage in cells treated with artesunate and ML385; Figure S4: ZIP model demonstrating synergy between artesunate and ML385; Figure S5: Uncropped Western blots.
