# Supplementary material for: KEAP1 Is Required for Artesunate Anticancer Activity in Non-Small-Cell Lung Cancer
Source: Cancers (Basel). 2021 Apr 14;13(8):1885. doi: 10.3390/cancers13081885 (PMC8070990; doi:10.3390/cancers13081885)
Supplement: Supplementary file 1 [file cancers-13-01885-s001.zip › Figure S5. original-images.pdf]

Uncropped Western blots for Figure 3

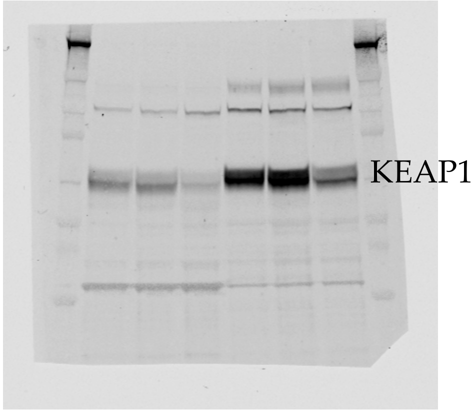

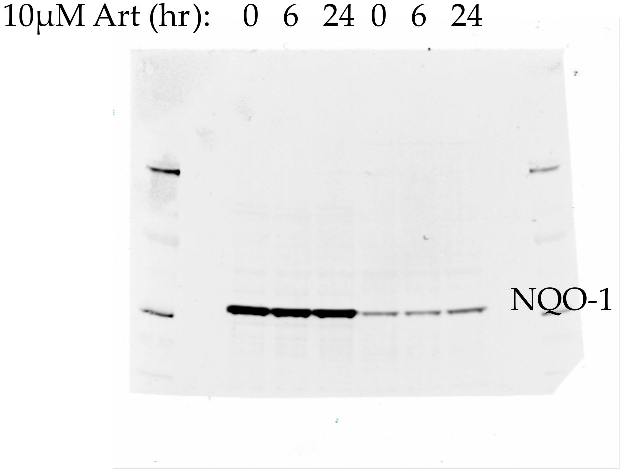

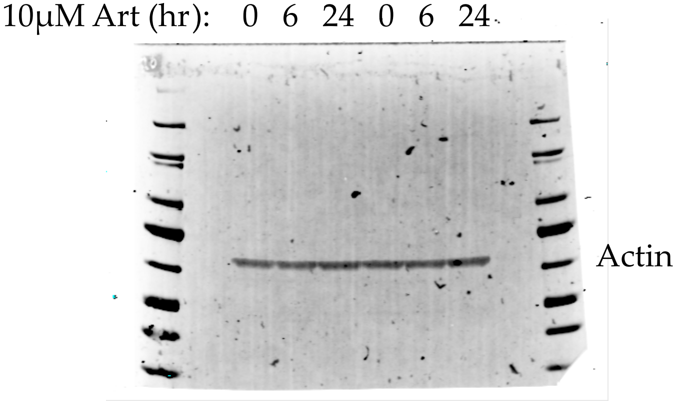

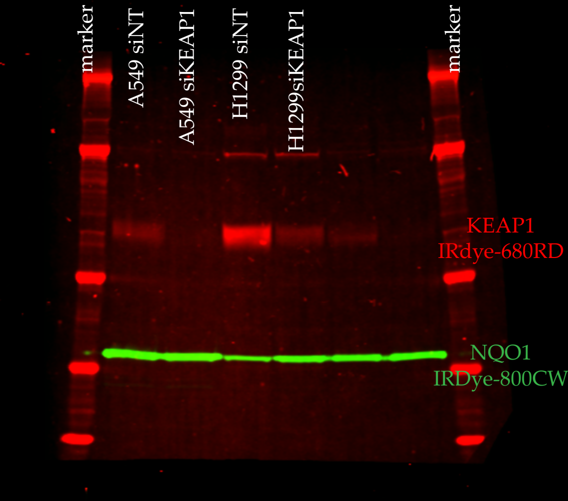

Uncropped Western blot for figure 4a

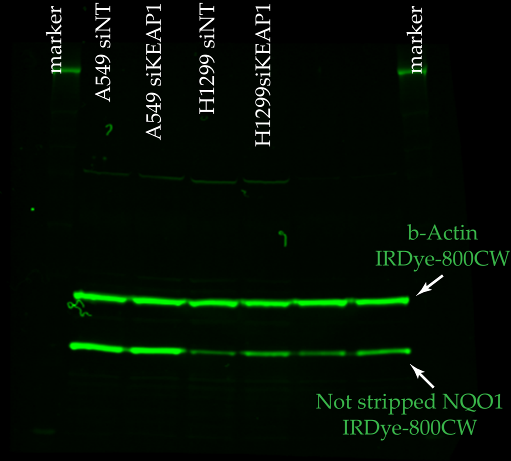

Uncropped Western blot for figure 4a
