# Supplementary material for: KEAP1 Is Required for Artesunate Anticancer Activity in Non-Small-Cell Lung Cancer
Source: Cancers (Basel). 2021 Apr 14;13(8):1885. doi: 10.3390/cancers13081885 (PMC8070990; doi:10.3390/cancers13081885)
Supplement: Supplementary file 1 [file cancers-13-01885-s001.zip › supplmentary figure S1-S4.pdf]

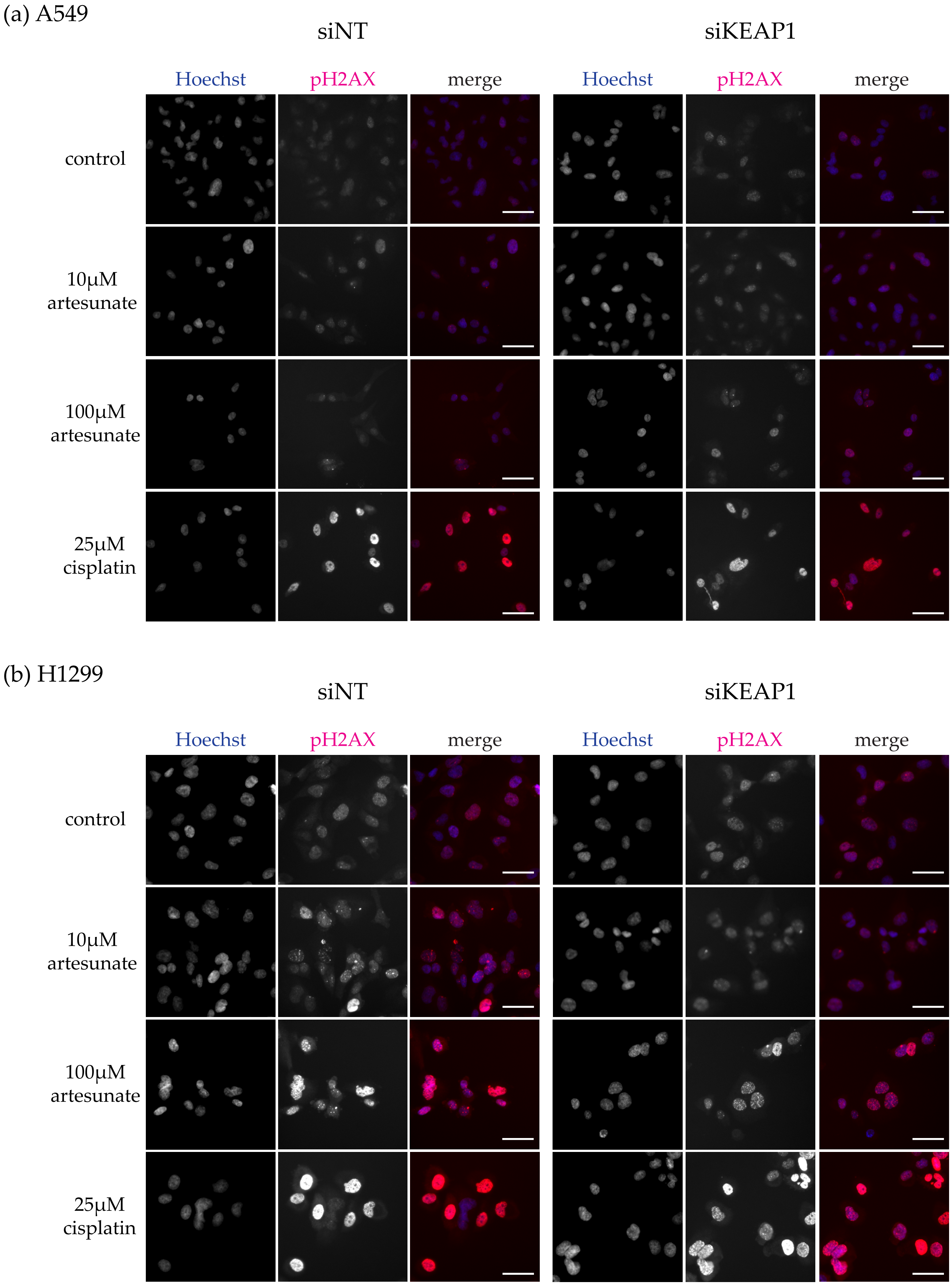

Supplemental Figure 1: Representative images of pH2AX staining in A549 (a) and H1299 cells (b) that had been transfected with non-targeting (siNT) or KEAP1 (siKEAP1) specific siRNA following 24 hour treatment with 0.1% DMSO (control), 10μM artesunate, 100μM artesuante, or 25μM cisplatin (bar = 50 μm).

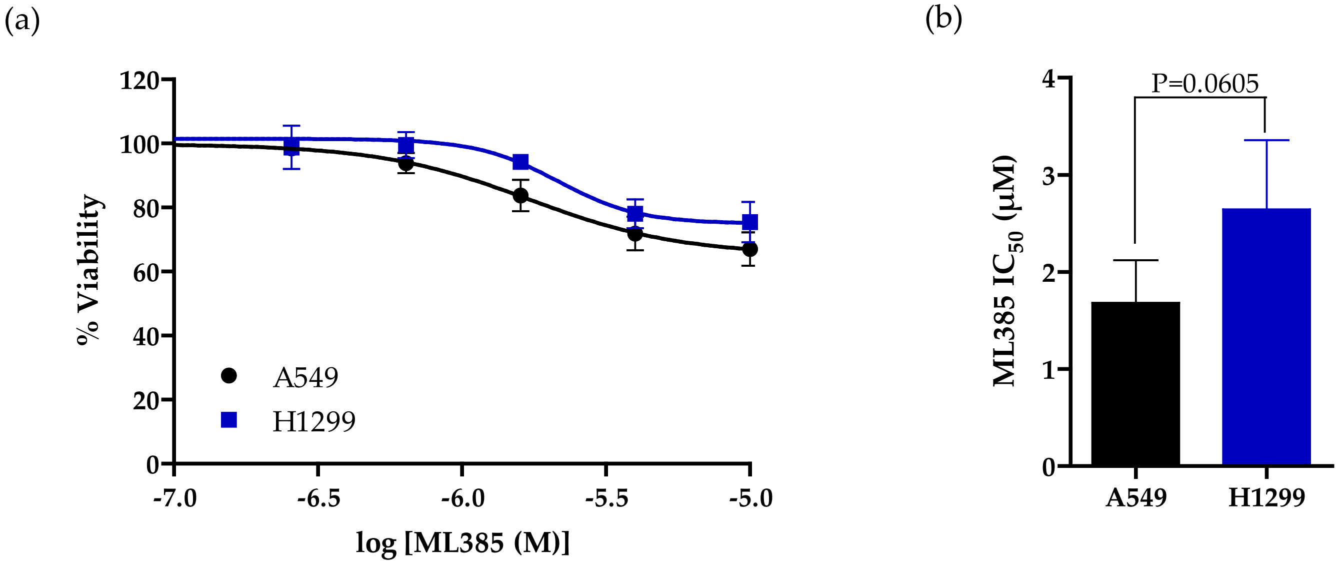

Supplemental Figure 2: ML385 (NRF2 inhibitor) has minimal effect of viability of A549 and H1299 NSCLC cell lines as a single agent (a) Cells were treated with a serially diluted concentrations of ML385 for 96hr. Each cell lines was normalized to cells treated with 0.1% DMSO as a control and is graphed as the mean +/- SD, n=4. (b) The mean IC50 of ML385 in each cell line is graphed +/- SD. P-value was calculated using a two-tailed t-test comparing the mean IC50 for each cell line tested.

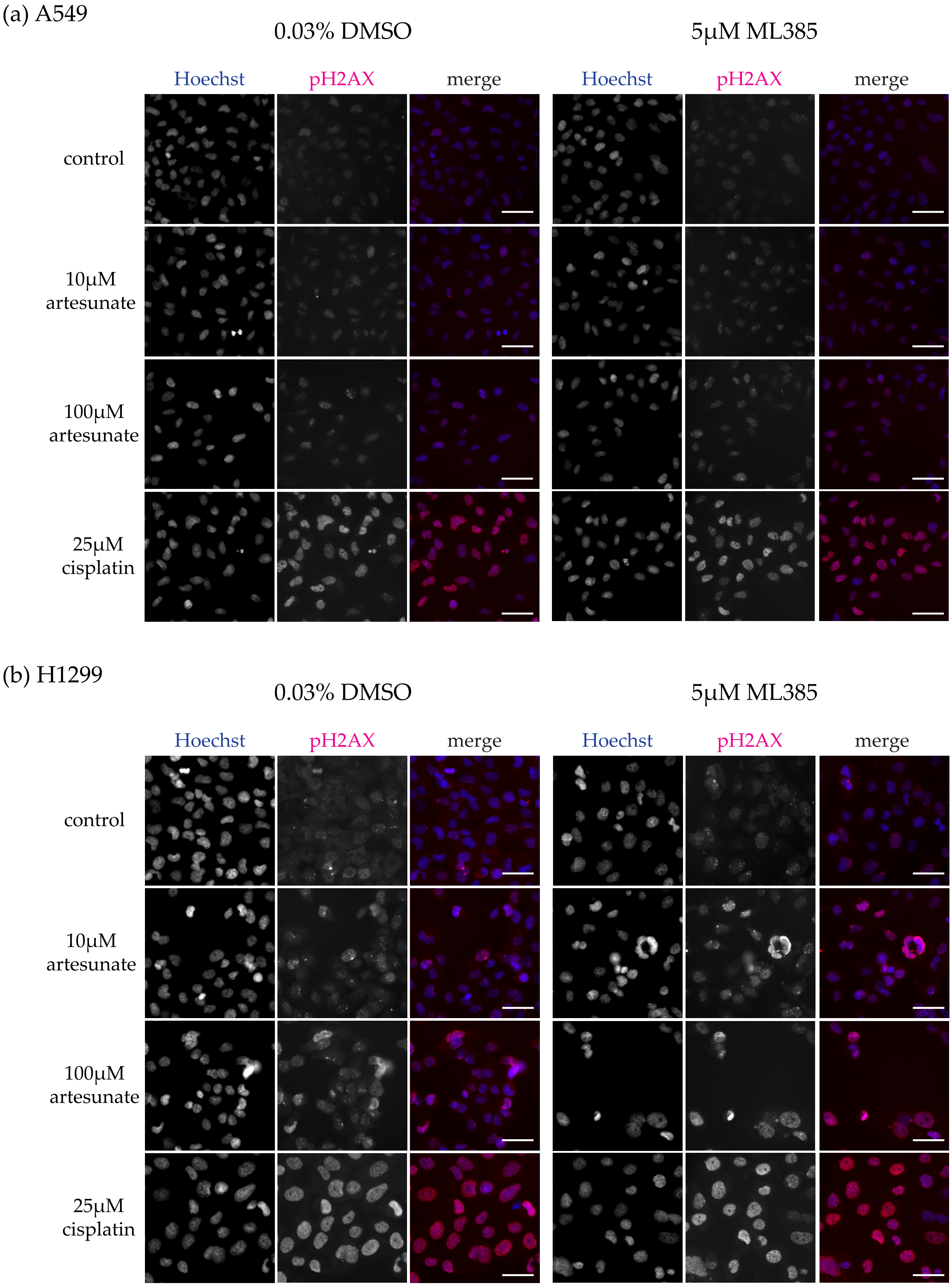

Supplemental Figure 3: Representative images of pH2AX staining in A549 (a) and H1299 cells (b) that had been treated with 0.03% DMSO or 5μM ML385 for 24 hour prior to treatment with 0.1% DMSO (control), 10μM artesunate, 100μM artesuante, or 25μM cisplatin for 24 hours(bar = 50 μm).

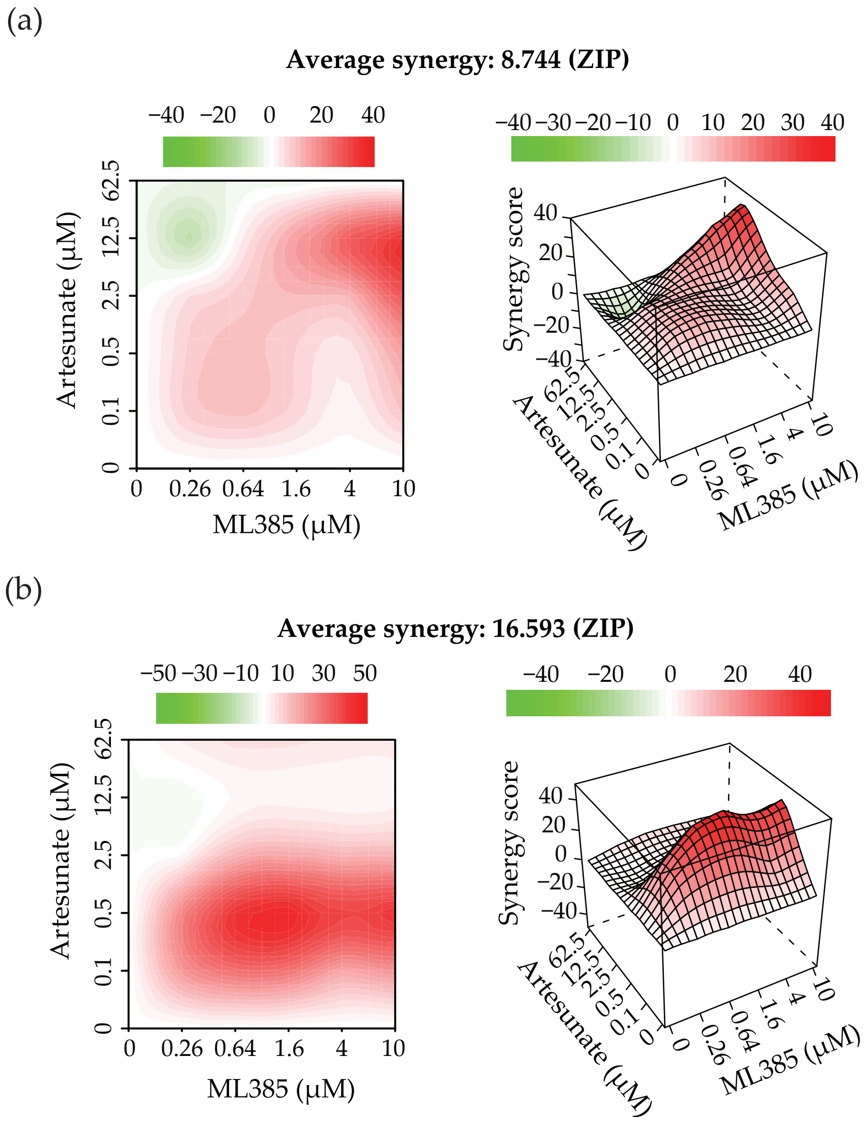

Supplemental Figure 4: ZIP model demonstrating synergy between artesunate and ML385
Graphic representation of the ZIP model of synergy scoring as calculated by using a 6x6 dose-response matrix in A549 (a) and H1299 (b) cells. Red color indicates synergy, while green indicates antagonism between the drug combinations tested.
